# Multivariate Trajectories of Weight and Mental Health and Their Prognostic Significance 6 Years After Obesity Surgery

**DOI:** 10.1002/eat.24527

**Published:** 2025-08-25

**Authors:** Anja Hilbert, Annika Strömer, Christian Staerk, Ben Schreglmann, Thomas Mansfeld, Johannes Sander, Florian Seyfried, Stefan Kaiser, Christine Stroh, Arne Dietrich, Ricarda Schmidt, Andreas Mayr

**Affiliations:** ^1^ Integrated Research and Treatment Center AdiposityDiseases, Behavioral Medicine Research Unit, Department of Psychosomatic Medicine and Psychotherapy University of Leipzig Medical Center Leipzig Germany; ^2^ Department of Medical Biometrics, Informatics and Epidemiology University Hospital Bonn Bonn Germany; ^3^ IUF—Leibniz Research Institute for Environmental Medicine Düsseldorf Germany; ^4^ Department of Statistics TU Dortmund University Dortmund Germany; ^5^ Department of General Surgery Asklepios Clinic Hamburg Germany; ^6^ Obesity Clinic Schön Klinik Hamburg Eilbek Hamburg Germany; ^7^ Department of General, Visceral, Transplant, Vascular and Pediatric Surgery University Hospital, University of Würzburg Würzburg Germany; ^8^ Department of Visceral, Pediatric and Vascular Surgery Hospital Konstanz Konstanz Germany; ^9^ Department of Obesity and Metabolic Surgery Municipal Hospital Gera Gera Germany; ^10^ Department of Surgery, Clinic for Visceral, Transplantation, Thoracic and Vascular Surgery University Hospital Leipzig Leipzig Germany

**Keywords:** bariatric surgery, multivariate trajectory modeling, psychopathology, quality of life, weight loss

## Abstract

**Objective:**

Obesity surgery (OS) results in substantial, albeit heterogeneous, long‐term improvements in weight and mental health, with unclear trajectories and their associations. This study examined multivariate trajectories of weight, psychopathology, and health‐related quality of life (HRQOL) after OS, and their prospective association with long‐term health outcomes.

**Method:**

In the prospective multicenter Psychosocial Registry of Obesity Surgery, *N* = 856 patients were classified into multivariate trajectory classes using latent class linear mixed models, based on assessments of weight, depression, eating disorder psychopathology, and HRQOL at baseline and annually 1–5 years following OS. The prognostic significance of trajectory classes for 6‐year follow‐up was examined. Multivariate trajectory modeling was compared with univariate weight trajectory modeling for concordance and prognostic significance.

**Results:**

We identified three trajectory classes of *low* (I, 2.8%), *medium* (II, 89.1%), and *high* (III, 8.1%) *sustainability* 1–5 years after OS, indicating high (I) or gradual deterioration (II) or further improvement (III) after initial improvement of indicators. The *low sustainability* class (I) reached nadir improvements earliest. Consistently, trajectory classes were prospectively associated with differential clinically significant improvement in weight and mental health at the 6‐year follow‐up. Multivariate trajectory modeling was discordant with univariate weight trajectory modeling and showed greater predictive value for health outcomes at the 6‐year follow‐up.

**Discussion:**

Patients who achieve nadir improvements in weight and mental health early may require clinical attention to prevent long‐term relapse. Monitoring changes in the first years after OS appears essential to identify patients in need of additional intervention, ideally using indicators beyond weight, such as mental health.

**Summary**

- This study analyzed combined physical and mental health changes over 5 years after obesity surgery, identifying three patient groups with low, medium, or high long‐term improvement.
- Considering multiple health indicators provided better outcome prediction than weight alone.
- Low improvement was linked to a higher risk of eating disorders.
- Monitoring of multiple factors appears crucial to identifying at‐risk patients needing extra care.
- Future research should explore these patterns over longer periods.

## 1 Introduction

Obesity surgery (OS) is currently the most efficacious and sustainable intervention for severe obesity (i.e., obesity class 3 body mass index [BMI] ≥ 40 kg/m^2^ or class 2 BMI ≥ 35 kg/m^2^ with obesity‐related comorbidities; National Institute for Health and Clinical Excellence 2006), an increasingly prevalent health disorder (Hales et al. 2018; Ward et al. 2019; Williamson et al. 2020). The commonly applied surgical procedures, laparoscopic Roux‐en‐Y gastric bypass (RYGB) and laparoscopic sleeve gastrectomy (SG), lead to a total weight loss of 20%–35% over 5–10 years of follow‐up (O'Brien et al. 2019; van Rijswijk et al. 2021), with weight regain mostly beginning around 2 years after reaching nadir weight. In the long term, a significant minority of patients experience poor weight loss (Arterburn et al. 2020) and less improvement of the adverse physical obesity‐related sequelae (e.g., type 2 diabetes mellitus; Adams et al. 2017; Courcoulas et al. 2018; Puzziferri et al. 2014).

Regarding mental health, OS produces substantial improvements in mental health, for example, in symptoms of depression (Dawes et al. 2016; Kalarchian et al. 2019) and eating disorders as well as health‐related quality of life (HRQOL; Andersen et al. 2015; Kalarchian et al. 2019; Kolotkin and Andersen 2017); however, deteriorations after initial improvement have been documented (Devlin et al. 2018; Hilbert et al. 2022; Kalarchian et al. 2019). Weight loss and improvements in mental health seem to co‐occur following OS (Hilbert et al. 2022; Nielsen et al. 2022), but their associations over time, prognostic relevance, and baseline correlates have yet to be explored (Hindle et al. 2017; Youssef et al. 2020). This is particularly relevant given the multifactorial nature of obesity presenting with physical and mental health‐related sequelae, including functional impairment at different severity levels (Sharma and Kushner 2009).

While available long‐term follow‐up studies usually report outcomes following OS cross‐sectionally by timepoints (Reis et al. 2023), initial studies have begun to explore post‐surgical weight trajectories through a longitudinal perspective from pretreatment over time. Using univariate person‐centered longitudinal latent structure analyses, prospective (Courcoulas et al. 2018; Slurink et al. 2024) and retrospective (Lent et al. 2018; Voorwinde et al. 2022) studies covering 3–7 years of follow‐up mostly identified three trajectories with varying degrees of weight loss and regain (range: 3–7 trajectories). This evidence was limited by a high loss to follow‐up and concentration on RYGB only, considering that weight trajectories following SG showed steeper weight regain (Shen et al. 2024). Only a few studies have addressed trajectories of mental health in addition to that of weight. For example, in their prospective linear growth mixture modeling of 3 years following RYGB (*N* = 420), Slurink et al. (2024) identified three different weight trajectories (high and moderate weight loss with weight regain, low weight loss without weight regain) and, separately, four or five trajectories of physical or mental HRQOL, respectively, similar to previous HRQOL modeling (Youssef et al. 2020). However, associations of HRQOL trajectories with weight trajectories were not analyzed. Using latent class growth mixture modeling on retrospective 5‐year data following OS, mainly RYGB (*N* = 2785), Voorwinde et al. (2022) identified five weight trajectories (average weight loss/fairly stable, above average weight loss/partial regain, low response, rapid weight loss/regain, continued weight loss). Separately, they modeled eating behavior and physical activity, resulting in three trajectories per variable that showed partial associations with weight trajectories.

Thus, while heterogeneity in weight trajectories after OS is increasingly being elucidated, their relation to HRQOL and mental health trajectories and their prognostic relevance remain unclear. Mechanistically, sustained weight loss following OS may involve long‐term improvements in HRQOL and mental health (Hilbert et al. 2022; Nielsen et al. 2022). In contrast, unresolved psychopathology at follow‐up—such as eating disorder symptoms and, less consistently, depression—was associated with reduced long‐term weight loss and weight regain (Devlin et al. 2018; Freire et al. 2021; Hilbert et al. 2022; Kalarchian et al. 2019), potentially by promoting increased energy intake or reduced physical activity. Postoperative eating disorder symptoms, depressive symptoms, and HRQOL have been found to be interrelated.

Overall, while changes in weight, HRQOL, and psychopathology often co‐occur after OS, it is uncertain whether their trajectories align, highlighting the need for multivariate rather than the previously applied univariate trajectory analyses. It is further largely unclear whether specific multivariate change trajectories have a differential prognostic relevance for long‐term physical and mental health outcomes and can improve identification of individuals at risk compared to univariate weight trajectory analysis. Thus, this study sought to investigate change trajectories in weight, psychopathology, and HRQOL following OS using multivariate trajectory analysis, allowing the identification of trajectory classes, their sociodemographic and clinical baseline correlates, and their prospective association with long‐term health outcomes. For sensitivity analysis, multivariate trajectory modeling was compared with univariate trajectory modeling of weight for concordance and prognostic significance.

## 2 Methods

### 2.1 Participants

This study is part of the ongoing prospective Psychosocial Registry for Obesity Surgery (PRAC) study (Hilbert et al. 2022) implemented at six surgical treatment centers in Germany upon approval by the local ethics committees and registration in the German Clinical Trials Register (DRKS00006749). Written informed consent was obtained from all patients prior to enrollment. The Strengthening the Reporting of Observational Studies in Epidemiology (STROBE) reporting guideline was followed for this study (von Elm et al. 2007).

Inclusion criteria were age ≥ 18 years and planned OS; exclusion criteria were lack of German language skills and noncompliance with study procedures. Study‐specific inclusion criteria consisted of the surgical procedures RYGB or SG; complete baseline data on the four indicator variables used for trajectory analysis; at least one follow‐up assessment 1–5 years following surgery; and enrollment between 03/2012 and 01/2023. Assessment time points were at baseline (T0, pre‐surgery) and 1–6 years (T2–T7) post‐operatively (T1 at 6 months not reported in this study because depression was not assessed).

A total of 1144 adult volunteers were enrolled in PRAC, of which 217 were excluded (surgery not received: 196; no RYGB or SG: 11; dropout before baseline: 10) leaving 927 eligible patients providing baseline data on the four trajectory variables, of whom *N* = 856 (92.34%) had at least one follow‐up assessment. During follow‐up, 17.29% (148/856; T2: 38; T3: 27; T4: 23; T5: 28; T6: 21; T7: 11) were lost due to withdrawal of consent (126/148) or death (22/148). From the total baseline sample of *N* = 856 (70.86%), 567 patients received RYGB (66.24%), 289 patients received SG (33.76%), and 2938 follow‐up assessments were available (T2: 754; T3: 629; T4: 533; T5: 438; T6: 329; T7: 255). Notably, in addition to study dropout (17.29%), further missingness resulted from the study's ongoing repeated‐measures data collection.

### 2.2 Measures for Multivariate Trajectory Analysis

Body weight and height were measured objectively using calibrated equipment (for details on the imputation of missing objective body weight data at follow‐up using subjective body weight, see the Data Analytic Plan). In addition to weight, three well‐established, validated measures were selected, covering different aspects of mental health with clinical relevance, based on a multidimensional definition of health (World Health Organization 1946). Depressive symptoms were assessed over the last 2 weeks by the 9‐item Patient Health Questionnaire (PHQ‐D; Gräfe et al. 2004; Spitzer et al. 1999) (0 = *not at all*; 3 = *almost every day*), with higher sum scores (0–27) indicative of a more severe level of depression (Cronbach's α = 0.85, 95% CI 0.82–0.86; McDonald's ω total = 0.88, 95% CI 0.80–0.99). Eating disorder psychopathology was assessed using the Eating Disorder Examination‐Questionnaire (EDE‐Q; Fairburn and Beglin 2008; Hilbert and Tuschen‐Caffier 2016), covering restraint, eating concern, weight concern, and shape concern with 22 items (0 = *characteristic was not present*; 6 = *characteristic was present every day/in extreme form*). A mean global score was derived, with higher scores indicating greater eating disorder psychopathology (Cronbach's α = 0.87, 95% CI 0.86–0.88; McDonald's ω total = 0.91, 95% CI 0.89–0.97). HRQOL was determined using the 31‐item Impact of Weight on Quality of Life‐Lite (IWQOL) questionnaire total sum score (Kolotkin et al. 2001; Mueller et al. 2011) (recoded as 0 = *worst* to 100 = *best*; Cronbach's α = 0.95, 95% CI 0.95–0.96; McDonald's ω total = 0.97, 95% CI 0.95–0.99). For correlations among the trajectory variables, see Table S1.

### 2.3 Outcome Measures

Outcomes were determined at T7 and included the continuous measures used for the trajectory analysis. In addition, established indicators of clinically significant change were used. For weight loss and clinically significant weight loss, percentage total body weight loss from baseline (%TBWL) and presence versus absence of %TBWL ≥ 20 were determined. In addition, percentage alterable weight loss (%AWL = 100 × (baseline BMI–follow‐up BMI)/(baseline BMI–13)) and presence versus absence of clinically significant %AWL ≥ 35 were calculated as new measures to estimate outcome independent of baseline BMI (van de Laar et al. 2018). For depressive symptoms, a PHQ‐D score < 10 versus ≥ 10 was used to determine absence versus presence of clinically significant, moderate depression (Gräfe et al. 2004). For eating disorder psychopathology, EDE‐Q global scores < 95^th^ percentile versus ≥ 95^th^ percentile of normative population means (Hilbert et al. 2012) were used as indicators for the absence versus presence of clinically significant eating disorder psychopathology. (For HRQOL, validated cut‐offs of meaningful change of IWQOL scores in obesity surgery or norms were unavailable.) In addition, adverse surgery‐related outcomes including bariatric reoperations and complications with the surgical procedure until T7, as well as improvement in obesity‐related comorbidities between baseline and T7, were assessed interview‐based using three items from the Bariatric Analysis and Reporting Outcome System (BAROS; Oria and Moorehead 2009) that were dichotomized (present, absent).

### 2.4 Baseline Correlates

Sex (male, female), age (years), education (low < 12 years, high ≥ 12 years of school education), and surgical procedure (RYGB, SG) were examined as baseline correlates of the multivariate trajectories; as were baseline scores of the continuous and dichotomous outcomes described above.

### 2.5 Data Analytic Plan

To derive multivariate trajectory classes in a data‐driven manner, latent class linear mixed models (LCMMs) were utilized to jointly model the longitudinal outcomes of weight, PHQ‐D, EDE‐Q, and IWQOL over the first 5 years after surgery. Weight was represented as relative weight loss to baseline over time (i.e., %TBWL), while PHQ‐D, EDE‐Q, and IWQOL scores were modeled as absolute differences (change scores) from baseline over time. If objective body weight was missing at follow‐up but subjective body weight was available, the objective body weight was imputed using linear regression, estimating the objective weight based on the subjective body weight. For all other outcomes, we used all available information at the corresponding timepoints in the linear mixed models without imputation. This approach is robust to missing data under various mechanisms, including the Missing at Random (MAR) assumption, and is well‐suited for prospective longitudinal designs (Twisk et al. 2013).

LCMMs were chosen to account for heterogeneity between patients by categorizing them into unobserved groups (latent trajectory classes) with distinct longitudinal class‐specific trajectories. Outcome‐ and class‐specific trajectories were modeled on all available data using flexible link functions based on splines, with random effects incorporated to adjust for repeated measurements and multiple outcomes. Model selection involved fitting models with varying numbers of latent classes and selecting the final number of classes based on minimizing the Akaike and Bayesian information criteria (AIC/BIC), maximizing entropy (i.e., class separation), and considering interpretation. Trajectory class membership of patients was determined based on posterior class probabilities. Mean and individual trajectories for all outcomes were graphically displayed, stratified by trajectory class.

To compare resulting classes from the LCMM regarding differences in baseline correlates and 6‐year outcomes, odds ratios (ORs) with two‐sided 95% confidence intervals (CIs) were calculated for categorical variables, and η^2^ measures with two‐sided 95% CIs were reported for continuous variables using all available data. Dunn's test was used for multiple post hoc comparisons. For sensitivity analysis, a univariate LCMM was computed solely for %TBWL, and the resulting classes were compared to those obtained from the LCMM for multiple outcomes. The comparison metric included predictive R^2^ values derived from linear models for each outcome with class as the predictor variable, as well as Cohen's κ and interclass correlation. Furthermore, mean and individual trajectories stratified by trajectory class were graphically displayed for all participants with available data at the final timepoint (T7) as well as for those with complete data across all timepoints (complete cases).

All statistical tests were conducted using two‐sided α = 0.05. Effect size interpretation was based on Cohen (small, medium, large: R^2^, 0.02, 0.13, 0.26; η^2^, 0.01, 0.06, 0.14; OR, 1.46, 2.50, 4.14; Chen et al. 2010; Cohen 1988). Analyses were performed via the statistical programming environment R version 4.0.4 and the lcmm add‐on package version 2.0.0.

## 3 Results

The sample of *N* = 856 patients in OS was predominantly middle‐aged, female, with a low level of education, presented with class 3 obesity, and underwent surgery at the University Medical Center Leipzig (Table 1). Across follow‐up, 13.32% (114/856) reported a bariatric reoperation.

**TABLE 1:** Sample characteristics.

	Total (*N* = 856)	Roux‐en‐Y gastric bypass (*N* = 567)	Sleeve gastrectomy (*N* = 289)
	No. (%)	No. (%)	No. (%)
Gender			
Female	587 (68.57%)	406 (71.60%)	181 (62.63%)
Male	269 (31.43%)	161 (28.40%)	108 (37.37%)
Age, years			
Median (IQR)	47.00 (37.00, 55.00)	48.00 (37.00, 55.00)	47.00 (36.00, 55.00)
Mean (SD)	46.39 (11.67)	46.61 (11.38)	45.97 (12.21)
Education^a^			
High	117 (16.98%)	74 (16.59%)	43 (17.70%)
Low	572 (83.02%)	372 (83.41%)	200 (82.30%)
Missing	167	121	46
Body weight, kg			
Median (IQR)	137.30 (122.40, 157.20)	133.80 (120.00, 149.20)	153.00 (128.30, 174.70)
Mean (SD)	141.56 (28.42)	135.60 (23.32)	153.80 (33.95)
Body mass index, kg/m^2^			
Median (IQR)	47.52 (42.55, 52.81)	46.41 (42.17, 50.69)	50.61 (43.44, 57.52)
Mean (SD)	48.40 (8.10)	46.41 (6.33)	51.68 (9.97)
Weight status^b^			
Obesity class 1	11 (1.29%)	10 (1.77%)	1 (0.35%)
Obesity class 2	100 (11.68%)	66 (11.68%)	32 (11.07%)
Obesity class 3	745 (87.23%)	489 (86.55%)	256 (88.58%)
Treatment center			
Leipzig	683 (79.79%)	471 (83.07%)	212 (73.36%)
Other	173 (21.21%)	96 (16.93%)	77 (26.64%)

*Note*: Zero missing values for each variable, with the exception of education.

Abbreviation: IQR, interquartile range.

^a^ School education: high, ≥ 12 years or higher; low, < 12 years.

^b^ Obesity class 1, body mass index 30.0–34.9 kg/m^2^; class 2, 35.0–39.9 kg/m^2^; class 3, ≥ 40.0 kg/m^2^.

### 3.1 Multivariate Trajectory Groups

**FIGURE 1:**
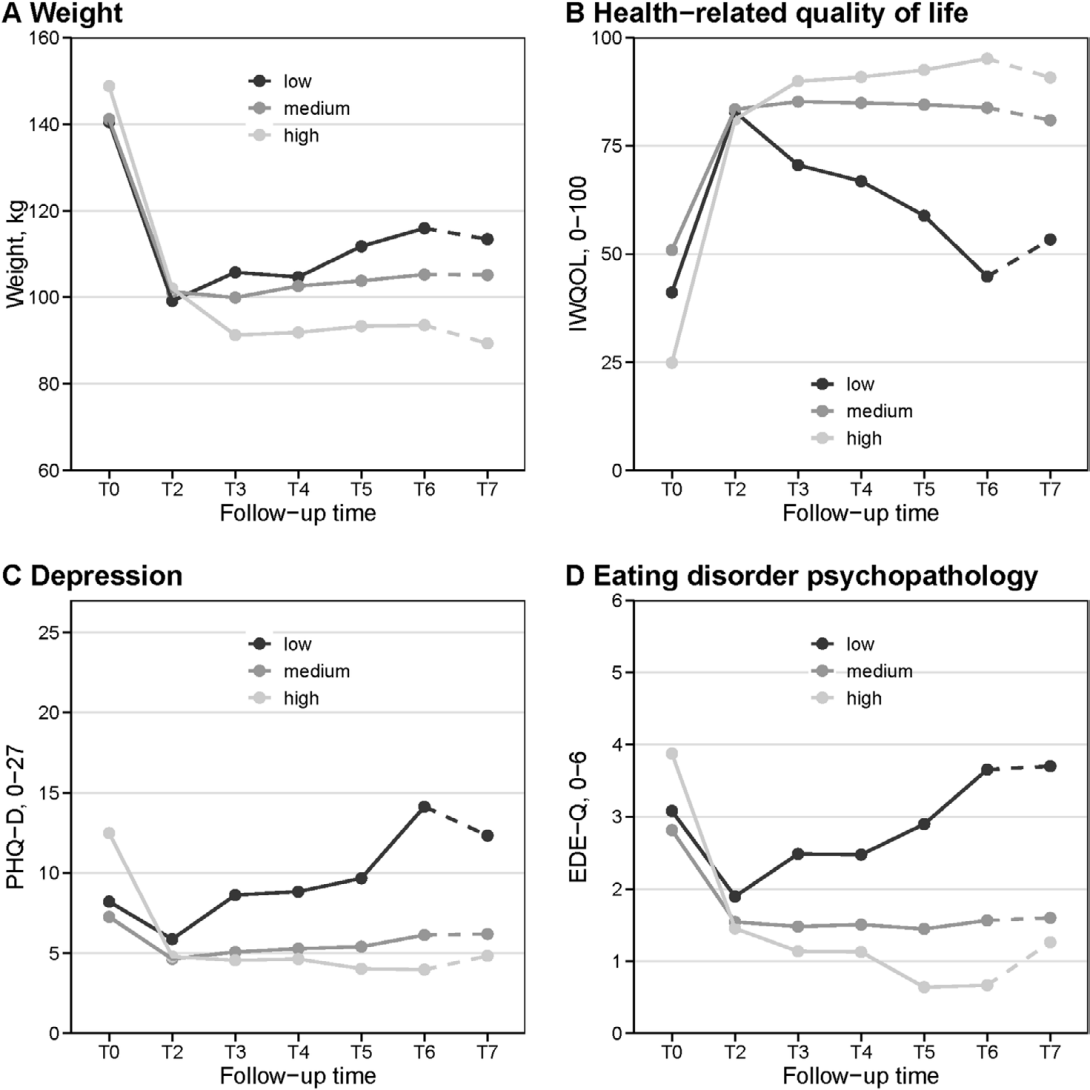
Multivariate trajectory classes of low, medium, and high sustainability determined via latent class linear mixture modeling conducted from baseline to the first 5 years after obesity surgery (T0–T6) and applied to the 6‐year follow‐up (T7; *N* = 856). Displayed are means from valid cases. EDE‐Q, Eating Disorder Examination‐Questionnaire; IWQOL, Impact of Weight on Quality of Life‐Lite; PHQ‐D, Patient Health Questionnaire‐Depression.

Data‐driven LCMM on changes in body weight, depression, eating disorder psychopathology, and HRQOL from T0 to T6 identified three distinct multivariate trajectory classes of *low* (I), *medium* (II), and *high* (III) *sustainability* (Figure 1; Table S2), reflecting early relapse (I), gradual deterioration (II), or continued improvement (III) after initial post‐surgical improvements from T0 to T2 (Table S3).

Descriptively, in the *low sustainability* class (I), 24 (2.80%) patients reached their nadir weight at T2, followed by a steep increase by T6. Similarly, their T2 postoperative improvement in depression, eating disorder psychopathology, and HRQOL deteriorated substantially by T6. The *medium sustainability* class (II) consisted of 763 (89.14%) patients who reached their nadir weight at T3 after a slight continued weight loss from T2 to T3, followed by a gradual weight regain from T2 to T6. Patients in this class maintained their T2 improvement in eating disorder psychopathology and HRQOL through T6, while experiencing a slight increase in depression. The *high sustainability* class (III) included 69 (8.06%) patients who reached their nadir weight at T3 after considerable sustained weight loss between T2 and T3. These patients showed the greatest improvements in all parameters at T2 and experienced further improvement in eating disorder psychopathology and HRQOL from T2 to T6, but a slight increase in depression during this time.

The multivariate trajectory classes identified in the total intent‐to‐treat sample showed a similar distribution and course in the subsample with available 6‐year follow‐up data (*N* = 225) and in the complete‐case subsample (*N* = 164; Figure S1). Figures S2–S4 illustrate the individual trajectories within each multivariate trajectory class for the total sample and the respective subsamples.

### 3.2 Baseline Correlates

Regarding baseline correlates, the trajectory classes did not differ significantly by sex, age, and education nor by weight or BMI (Table 2; mostly less than small to small effects). However, they differed in mental health (medium to large effects): Depressive symptoms and eating disorder psychopathology, and clinically significant levels thereof, were lower in the *low* and *medium sustainability* classes than in the *high sustainability* class, and HRQOL was lower in the *high sustainability* class than in the *low sustainability* class, whereas the *medium sustainability* class had higher HRQOL than the other two classes.

**TABLE 2:** Baseline sociodemographic and clinical correlates and 6‐year clinical outcomes of multivariate trajectories of low, medium, and high sustainability (*N* = 856).

	Class 1: Low sustainability (*n* = 24)	Class 2: Medium sustainability (*n* = 763)	Class 3: High sustainability (*n* = 69)	Effect size
	No. (%) or mean (SD)	No. (%) or mean (SD)	No. (%) or mean (SD)	
**Baseline correlates**				
Sex, female	16 (66.67%)	519 (68.02%)	52 (75.36%)	1 vs. 2: OR = 1.06, 95% CI, 0.39 to 2.68 3 vs. 2: OR = 0.70, 95% CI, 0.37 to 1.25 1 vs. 3: OR = 0.66, 95% CI, 0.22 to 2.10
Sex, male	8 (33.33%)	244 (31.98%)	17 (24.64%)	
Age, years	44.33 (10.45)	46.39 (11.84)	47.07 (10.09)	η^2^ = 1.15e‐03, 95% CI, 0.00 to 0.01
Education, low	16 (76.19%)	501 (82.95%)	55 (85.94%)	1 vs. 2: OR = 1.52, 95% CI, 0.43 to 4.46 3 vs. 2: OR = 0.80, 95% CI, 0.34 to 1.69 1 vs. 3: OR = 0.53, 95% CI, 0.13 to 2.30
Education, high	5 (23.81%)	103 (17.05%)	9 (14.06%)	
Education total	21	604	64	
Education missing	3	159	5	
Body weight, kg	140.50 (25.46)	141.15 (28.40)	148.78 (32.09)	η^2^ = 5.30e‐03, 95% CI, 0.00 to 0.02
Body mass index, kg/m^2^	47.81 (8.03)	41.14 (8.01)	51.45 (8.49)	η^2^ = 0.01, 95% CI, 0.00 to 0.03
PHQ‐D, 0–27	8.21 (5.30)^a^	7.26 (4.93)^a^	12.49 (4.70)^b^	η^2^ = 0.08, 95% CI, 0.05 to 0.12
PHQ‐D ≥ 10	8 (33.33%)	188 (27.49%)	51 (73.91%)	1 vs. 2: OR = 1.32, 95% CI, 0.48 to 3.33 3 vs. 2: OR = 7.45, 95% CI, 4.15 to 13.92 1 vs. 3: OR = 5.54, 95% CI, 1.87 to 17.77
PHQ‐D total (*N* = 777)	24	684	69	
PHQ‐D missing	0	79	0	
EDE‐Q, 0–6	3.08 (0.96)^a^	2.82 (0.98)^a^	3.88 (0.84)^b^	η^2^ = 0.09, 95% CI, 0.05 to 0.13
EDE‐Q ≥ 95%	18 (75.00%)	469 (68.37%)	66 (95.65%)	1 vs. 2: OR = 1.39, 95% CI, 0.52 to 4.33 3 vs. 2: OR = 10.16, 95% CI, 3.27 to 51.10 1 vs. 3: OR = 7.13, 95% CI, 1.37 to 48.36
EDE‐Q total (*N* = 779)	24	686	69	
EDE‐Q missing	0	77	0	
IWQOL, 0–100	41.12 (19.91)^a^	50.89 (21.14)^b^	24.87 (13.08)^c^	η^2^ = 0.12, 95% CI, 0.08 to 0.16
IWQOL total (*N* = 778)	24	685	69	
IWQOL missing	0	78	0	
**Surgical correlates**				
Surgical procedure, RYGB	14 (58.33%)	509 (66.71%)	44 (63.77%)	1 vs. 2: OR = 1.43, 95% CI, 0.56 to 3.52 3 vs. 2: OR = 1.14, 95% CI, 0.65 to 1.95 1 vs. 3: OR = 0.80, 95% CI, 0.28 to 2.33
Surgical procedure, SG	10 (41.67%)	254 (33.29%)	25 (36.23%)	
Reoperations over 6 years	5 (20.83%)	99 (12.98%)	10 (14.49%)	1 vs. 2: OR = 2.37, 95% CI, 0.50 to 5.03 3 vs. 2: OR = 1.14, 95% CI, 0.50 to 2.34 1 vs. 3: OR = 0.65, 95% CI, 0.17 to 2.72
Complications over 6 years	6 (27.27%)	160 (32.27%)	16 (32.00%)	1 vs. 2: OR = 0.77, 95% CI, 0.24 to 2.13 3 vs. 2: OR = 0.97, 95% CI, 0.48 to 1.86 1 vs. 3: OR = 1.25, 95% CI, 0.37 to 4.66
Complications total (*N* = 561)	22	489	50	
Complications missing	2	274	19	
**Clinical outcomes at 6 years**				
Body weight, kg	113.42 (22.04)^a^	105.16 (23.06)^a^	89.34 (18.65)^b^	η^2^ = 0.05, 95% CI, 0.01 to 0.10
%TBWL	21.03 (11.37)^a^	25.03 (10.90)^a^	35.40 (10.75)^b^	η^2^ = 0.08, 95% CI, 0.02 to 0.14
%TBWL ≥ 20%	4 (40.00%)	149 (67.12%)	22 (95.65%)	1 vs. 2: OR = 0.33, 95% CI, 0.07 to 1.43 3 vs. 2: OR = 10.72, 95% CI, 1.67 to 449.96 1 vs. 3: OR = 28.06, 95% CI, 2.51 to 1564.35
Body mass index, kg/m^2^	38.88 (6.85)^a^	35.89 (7.03)^a^	31.92 (6.40)^a^	η^2^ = 0.03, 95% CI, 0.00 to 0.09
%AWL	28.49 (14.66)^a^	34.50 (14.93)^a^	48.26 (13.93)^b^	η^2^ = 0.07, 95% CI, 0.02 to 0.14
%AWL ≥ 35%	2 (20.00%)	109 (49.10%)	19 (82.61%)	1 vs. 2: OR = 0.26, 95% CI, 0.03 to 1.35 3 vs. 2: OR = 4.90, 95% CI, 1.56 to 20.42 1 vs. 3: OR = 16.73, 95% CI 2.27 to 220.65
Body weight total (*N* = 255)	10	222	23	
Body weight missing	14	541	46	
PHQ‐D, 0–27	12.33 (6.42)^a^	6.19 (5.53)^a^	4.83 (3.45)^b^	η^2^ = 0.18, 95% CI, 0.10 to 0.27
PHQ‐D ≥ 10	4 (44.44%)	48 (25.13%)	2 (8.70%)	1 vs. 2: OR = 2.37, 95% CI, 0.45 to 11.52 3 vs. 2: OR = 0.28, 95% CI, 0.03 to 1.24 1 vs. 3: OR = 0.13, 95% CI, 0.01 to 1.19
PHQ‐D total (*N* = 223)	9	191	23	
PHQ‐D missing	15	572	46	
EDE‐Q, 0–6	3.70 (1.58)^a^	1.60 (1.26)^b^	1.26 (0.96)^c^	η^2^ = 0.18, 95% CI, 0.09 to 0.27
EDE‐Q ≥ 95%	7 (77.78%)	59 (30.57%)	3 (13.04%)	1 vs. 2: OR = 7.86, 95% CI, 1.55 to 79.71 3 vs. 2: OR = 0.34, 95% CI, 0.06 to 1.22 1 vs. 3: OR = 0.05, 95% CI, 0 to 0.41
EDE‐Q total (*N* = 225)	9	193	23	
EDE‐Q missing	15	570	46	
IWQOL, 0–100	53.36 (25.85)^a^	80.92 (19.93)^a^	90.76 (13.33)^b^	η^2^ = 0.26, 95% CI, 0.16 to 0.34
IWQOL total (*N* = 224)	9	192	23	
IWQOL missing	15	571	46	
Somatic comorbidity, improved	6 (75.00%)	170 (85.43%)	19 (100%)	1 vs. 2: OR = 0.51, 95% CI, 0.09 to 5.44
Somatic comorbidity total (*N* = 226)	8	199	19	
Somatic comorbidity missing	16	564	50	

*Note*: Zero missing values for sex, age, surgical procedure, and reoperations.

Abbreviations: %AWL, percentage alterable weight loss; %TBWL, percentage total body weight loss; EDE‐Q, Eating Disorder Examination‐Questionnaire; IWQOL, Impact of Weight on Quality of Life‐Lite; PHQ‐D, Patient Health Questionnaire‐Depression; RYGB, Roux‐en‐Y gastric bypass; SG, sleeve gastrectomy.

*p* < 0.05; The superscript letters a, b, c indicate significant Dunn's posthoc tests.

### 3.3 Clinical Outcomes at 6‐Year Follow‐Up

When prospectively relating trajectory classes derived from T0 to T6 to weight loss outcome at T7, %TBWL and %AWL and their dichotomized variants %TBWL ≥ 20% and %AWL ≥ 35% consistently differed by class and were significantly lower in the *low* and *medium sustainability* classes than in the *high sustainability* class, while weight and BMI were higher (Table 2; mostly medium to large effects).

Regarding psychological outcomes, trajectory classes differentially related to depressive symptoms and HRQOL (large effects), but not to clinically significant, moderate depression (PHQ‐D ≥ 10) at T7 (less than small to small effects); these outcomes were more favorable in the *high sustainability* class than in the *medium* and *low sustainability* classes (Table 2). Eating disorder psychopathology differed among all trajectory classes (large effect), with higher scores in those with *low* than *medium sustainability* and in those with *medium* than *high sustainability*. A clinically significant eating disorder psychopathology (EDE‐Q ≥ 95^th^ percentile) at T7 was more likely in the *low* than *medium* and *high sustainability* classes (large or small effect, respectively), whereas the differences in the *medium* and *high sustainability* classes did not reach statistical significance (less than small effect). Trajectory classes did not differ significantly in patient‐reported improvement of physical comorbidity from baseline through T7 (less than small effect). Descriptively, the proportion of patients reporting improvement was a quarter higher in the *high* than *low sustainability* class.

### 3.4 Surgical Correlates

Trajectory classes were unrelated to surgical procedure, bariatric reoperations, or complications following the initial operation up to T7 (Table 2; mostly less than small effects). When the 3 trajectory classes were applied separately to RYGB and SG patients, a similar distribution of patients in the *low*, *medium*, and *high sustainability* classes was found (RYGB: 14, 2.47%; 509, 89.77%; 44, 7.76%; SG: 10, 3.46%; 254, 87.89%; 25, 8.65%). Descriptively, the most notable difference occurred in the weight trajectory following SG (Figure S5): the *low sustainability* class had the lowest nadir weight, but steepest weight regain.

### 3.5 Sensitivity Analysis: Univariate LCMM for Weight

A univariate LCMM for relative weight loss from baseline only (i.e., %TBWL) also identified three distinct trajectory classes from T0–T6, with a *low sustainability* class including 7 (0.84%) patients, a *medium sustainability* class including 826 (98.92%) patients, and a *high sustainability* class including 2 (0.24%) patients. The univariate and the multivariate class solutions were neither concordant nor correlated in their classification of patients with values close to zero (κ = 0.02, z = 0.93, *p* = 0.35; intraclass correlation coefficient = 0.04, *p* = 0.15). The univariate model explained significantly less variance in the prediction of clinical outcomes at 6‐year follow‐up (T7) than the multivariate model in %TBWL (predictive R^2^: 0.16% vs. 7.64%), depressive symptoms (predictive R^2^: 2.32% vs. 18.42%), eating disorder psychopathology (predictive R^2^: 0.08% vs. 18.00%), and HRQOL (predictive R^2^: 0.65% vs. 25.54%; *p* < 0.001). Thus, the univariate model mostly involved less than small predictive effects across outcomes including %TBWL, whereas the multivariate model mostly involved moderately sized predictive effects.

## 4 Discussion

OS results in substantial improvements in weight and associated sequelae, but many patients face challenges with weight recidivism, relapse in psychopathology, and HRQOL impairment over the long term. The contributing factors are likely multifactorial, but not well understood, and may be mutually reinforcing. However, research on long‐term outcomes after OS has traditionally focused on single variables, particularly weight, examined cross‐sectionally as separate snapshots at single time points or, more recently, in emerging weight trajectory analyses. An advantage of the multivariate trajectory modeling used in this study is that longitudinal data on multiple clinically relevant variables were considered jointly to generate individual trajectories grouped into latent trajectory classes, allowing an understanding of how different outcomes change postsurgically in relation to each other. Importantly, the identified multivariate trajectory class solution derived over 5 years of follow‐up was found to be superior in predicting clinical outcomes, including weight at 6 years of follow‐up, compared to discordant univariate weight trajectory modeling. It was unrelated to baseline weight and inversely related to baseline mental health.

Using multivariate latent class linear mixed modeling, we identified three latent trajectory classes, similar to previous research on univariate weight trajectories (Lent et al. 2018; Shen et al. 2024; Slurink et al. 2024). Descriptively, patients in all trajectory classes achieved similar levels of improvement at 1 year after OS, but exhibited specific trajectories thereafter, showing: *low sustainability* (I), including weight regain, relapse beyond baseline levels of depression and eating disorder psychopathology, and HRQOL impairment; *medium sustainability* (II), with slight weight regain and maintenance or slight deterioration in psychopathology and HRQOL; and *high sustainability* (III), with slight weight regain, but continued improvement in psychopathology and HRQOL. Notably, individual trajectories within the medium sustainability class showed within‐class variability (see Figure S2), which is common in latent trajectory modeling and reflects the clinical diversity typically observed in large patient groups, thus warranting caution in interpreting mean trends. Taken together, the results suggest that different indicators do not change in parallel, but show distinctive courses, with most similarity of change in the nonindependent psychopathological and social impairment variables (Table S1).

Consistent with previous weight trajectory analyses spanning 5–7 years following RYGB, the vast majority (~90%) of patients showed moderate long‐term weight loss and some degree of weight regain (Courcoulas et al. 2018; Voorwinde et al. 2022). Although patients were assigned to multiple classes rather than one class with *medium sustainability*, this pattern lends support to the validity of our multivariate trajectory class distribution. In addition, these previous studies also identified two smaller trajectory classes, comprising approximately 10% of patients, characterized by a low weight loss response or sustained high weight loss, resembling our *low* and *high sustainability* classes, respectively.

Further consistent with most previous weight trajectory analyses (Courcoulas et al. 2018; Lent et al. 2018; Shen et al. 2024; Voorwinde et al. 2022), a later nadir weight was associated with greater long‐term weight loss. While patients with a *low sustainability* trajectory reached the nadir of all indicators at 1‐year follow‐up, patients with a *medium sustainability* trajectory reached the nadir of all indicators at 2‐year follow‐up. In contrast, patients with a *high sustainability* trajectory reached their weight nadir at 2‐year follow‐up, while their psychopathology and HRQOL continued to improve throughout follow‐up. Ultimately, the trajectories resulted in low, medium, and high improvements at 5‐year follow‐up, in line with weight outcomes in univariate weight trajectory modeling (Courcoulas et al. 2018; Lent et al. 2018; Shen et al. 2024; Voorwinde et al. 2022), suggesting that the timepoint of maximal improvement of multiple indicators is critical to identify the trajectory class within which a patient belongs. A comparison of the trajectory classes by surgical procedures supported that the weight at nadir may be less critical for determining long‐term success than the timing; in fact, after SG, the *low sustainability* class achieved the lowest nadir weight of all trajectory groups at 1‐year follow‐up, followed by the steepest weight regain (Shen et al. 2024). Notable also was that the *high sustainability* class reached the largest improvements at 5 years across surgical procedures, although these patients had displayed worst baseline levels in indicators of mental health, also found in some (Courcoulas et al. 2018; Lent et al. 2018) but not other (Shen et al. 2024; Voorwinde et al. 2022) univariate weight trajectory modeling studies.

Predictor analyses supported these descriptive results at 6‐year follow‐up, suggesting prognostic relevance of the trajectory classes for long‐term weight, BMI, %TBWL, %AWL, depression, eating disorder psychopathology, and HRQOL with mostly medium to large effect sizes. Furthermore, they highlighted differences in clinically significant improvement, for example, of %TBWL ≥ 20% or %AWL ≥ 35% (van de Laar et al. 2018), and eating disorder psychopathology below clinical cut‐offs at 6 years following OS, especially when comparing the *high sustainability* class to the other classes with up to large effect sizes. Although most outcomes descriptively showed highest impairment in the *low sustainability* trajectory (*n* = 24), this class did not differ statistically from the *medium sustainability* trajectory, except for (clinically significant) eating disorder psychopathology with large effect sizes. Thus, the *low sustainability* class was especially characterized by elevated eating disorder psychopathology at 6‐year follow‐up. These results suggest that the multivariate trajectory modeling in our study has prognostic significance, which is particularly relevant in light of the inconsistent evidence on long‐term outcome prediction after OS (El Ansari and Elhag 2021), although the small size of the *low* and *high sustainability* classes underscores the need for replication of the multivariate trajectory modeling in independent samples. In addition, the stability of the findings and predictive validity should be tested over a longer time period. The increase in eating disorder psychopathology over follow‐up in the *low sustainability* class permits speculation that the majority of patients in this class (re)developed an eating disorder syndrome after OS, which may have impaired long‐term weight maintenance (Devlin et al. 2018; Hilbert et al. 2022).

Regarding predictors of trajectory classes, although baseline sociodemographic correlates and surgical procedures were not prospectively associated with trajectory class membership, several patterns warrant note: Descriptively, the *low sustainability* class included the highest proportion of individuals with high education and patients receiving SG, whereas the *high sustainability* class included the highest proportion of female patients and individuals with low education, aspects previously associated with unfavorable versus favorable weight trajectories (Courcoulas et al. 2018; Keith Jr et al. 2018; Lent et al. 2018; Shen et al. 2024). Trajectory classes were unrelated to baseline weight or BMI but showed a medium‐sized inverse relationship with psychopathological and social impairments, with the highest baseline impairments in the *high sustainability* class, which included the largest proportion of patients with clinically significant depression and eating disorder psychopathology. Thus, OS may have yielded the greatest relief from impairments alongside the most substantial and sustained weight loss, although causal inference requires experimental designs with control for confounding. This effect may partly reflect the exclusion of patients with unstable psychological conditions (e.g., severe mental disorders) from OS, in line with evidence‐based obesity treatment guidelines (Deutsche Adipositas Gesellschaft 2024).

Further, the divergence in psychological symptom trajectories despite similar weight regain between the *low* and *medium sustainability* classes suggests that factors beyond weight regain have contributed to increased depressive and disordered eating symptoms over time. Potential contributors include psychosocial stressors (e.g., postsurgical lifestyle adjustment difficulties), maladaptive coping (e.g., emotional eating), or adverse life events (e.g., separation). Moreover, preexisting vulnerabilities (e.g., low self‐esteem) may not have been fully captured at baseline. Future research should investigate diverse psychosocial factors over time to elucidate postsurgical trajectories. Of note, trajectory classes did not differ significantly by patient‐reported complications following surgery and revisional surgery. Previous research had found an association of revisional surgery with weight outcomes, but not with mental health outcomes (Courcoulas et al. 2018; Eisenberg et al. 2023; Leung et al. 2023).

Regarding strengths and limitations, this study was based on the multicenter PRAC study's large prospective cohort of adults with severe obesity undergoing RYGB and SG as standard surgical procedures (Angrisani et al. 2021). For the first time, a multivariate instead of univariate trajectory analysis approach was used, with trajectory variables being selected based on their relevance for physical, psychopathological, and social aspects of health in persons with severe obesity mapping onto the World Health Organization's multidimensional health definition (World Health Organization 1946). We used data from baseline through the first 5 years after OS to derive a trajectory class solution with long‐term prognostic significance. Of note, from baseline through 2–4‐year follow‐up, three‐class solutions were found, but with different composition and lower stability (data not shown), underscoring the importance of examining multi‐year postoperative trajectories to capture patterns of weight regain beyond initial weight loss (Pyykkö et al. 2025). Minimal selection, information, and confounding bias, and a high degree of generalizability to OS populations were ensured by minimal inclusion and exclusion criteria and well‐established assessments.

Attrition was relatively low (17.29%) and within the range commonly reported in psychosocial follow‐up studies with similar designs (10%–35% for RYGB; Devlin et al. 2018; Lent et al. 2018). Missing data were accounted for by longitudinal mixed‐effects modeling, which is robust to missing data, thereby reducing a potential attrition bias. Notwithstanding, the decrease in the analyzed sample from 856 to 329 at 5 years and 255 at 6 years likely contributed to the graphical variations of the trajectories from T6 to T7 (Figure 1) and made split‐sample LCMM analyses for SG and RYGB separately infeasible; therefore, the total sample LCMM was applied to the SG and RYGB subsamples (Figure S5). Trajectory classes in the 6‐year follow‐up and complete‐case subsamples closely resembled those in the total sample, supporting the robustness of our findings despite missing data. Regarding further limitations, ethnicity was not assessed due to cultural norms in Germany. Finally, it should be noted that instruments such as the PHQ‐D, while widely used in obesity research, were not developed or normed specifically for bariatric populations. This may lead to misinterpretation of obesity‐related symptoms, particularly those concerning sleep, eating behavior, or physical activity.

Future research should examine multivariate trajectories and their prognostic significance over a longer‐term follow‐up period with greater trajectory class sizes. Given the observed heterogeneity in individual courses within trajectory classes, especially the medium sustainability class, future studies may benefit from analytic approaches that capture within‐class variability and intraindividual dynamics more explicitly. Other indicators with potential clinical relevance may be considered to further our understanding of postsurgical change across multiple symptoms (e.g., self‐regulation, eating behavior, physical activity, hormonal changes; Schäfer et al. 2019). Although LCMM has limited value for identification of individuals at risk in clinical practice, our results support that patients who achieve nadir improvements in weight and mental health early may require clinical attention to prevent long‐term relapse and aggravation. A more fine‐grained analysis of change during the honeymoon phase of initial weight loss following OS may add specificity to this study's results. Clinically, monitoring change during the first years after OS appears essential to identify those in need of additional intervention, ideally using multiple established indicators, including those of mental health, as consideration of weight alone revealed inferior prognostic significance.

## Author Contributions

**Anja Hilbert:** conceptualization, funding acquisition, writing – original draft, writing – review and editing. **Annika Strömer:** conceptualization, formal analysis, writing – original draft, writing – review and editing. **Christian Staerk:** conceptualization, formal analysis, writing – original draft, writing – review and editing. **Ben Schreglmann:** investigation, writing – review and editing. **Thomas Mansfeld:** investigation, writing – review and editing. **Johannes Sander:** investigation, writing – review and editing. **Florian Seyfried:** investigation, writing – review and editing. **Stefan Kaiser:** investigation, writing – review and editing. **Christine Stroh:** investigation, writing – review and editing. **Arne Dietrich:** investigation, writing – review and editing. **Ricarda Schmidt:** investigation, writing – original draft, writing – review and editing. **Andreas Mayr:** conceptualization, formal analysis, writing – original draft, writing – review and editing.

## Ethics Statement

Ethical approval was granted by the Ethics Committee of Leipzig University Medical Center (Ref. No. 356‐11), based on which approval was granted by site‐specific Institutional Review Boards. Informed written consent was obtained at the outset of the study. The authors assert that all procedures contributing to this work comply with the ethical standards of the relevant national and institutional committees on human experimentation and with the Helsinki Declaration of 1975, as revised in 2008.

## Conflicts of Interest

Dr. Hilbert reports receiving research grants from the German Federal Ministry of Education and Research, German Research Foundation, Innovation Fund, and Roland Ernst Foundation for Health Care; royalties for books on the treatment of eating disorders and obesity with Hogrefe and Kohlhammer; honoraria for workshops and lectures on eating disorders and obesity and their treatment, including from Lilly and Novo Nordisk; honoraria as editor of the *International Journal of Eating Disorders*; and honoraria as a consultant for Takeda.

## Supporting information


**Data S1:** Supporting Information.

## Data Availability

The data that support the findings of this study are available on reasonable request from the corresponding author. The data are not publicly available due to privacy or ethical restrictions.
