# Supplementary material for: Multivariate Trajectories of Weight and Mental Health and Their Prognostic Significance 6 Years After Obesity Surgery
Source: Int J Eat Disord. 2025 Aug 25;58(11):2214–26. doi: 10.1002/eat.24527 (PMC12614669; doi:10.1002/eat.24527)
Supplement: Supplementary file 1 — Data S1: Supporting Information. [file EAT-58-2214-s001.pdf]

Multivariate trajectories of weight and mental health and prognostic significance over six years after obesity surgery

Anja Hilbert, Annika Strömer, Christian Staerk, Ben Schreglmann, Thomas Mansfeld, Johannes Sander, Florian Seyfried, Stefan Kaiser, Christine Stroh, Arne Dietrich, Ricarda Schmidt, & Andreas Mayr

Online Supplementary Material

Supplemental Tables

Table S1. Correlations Among Trajectory Variables at Baseline (N=856).

Table S2. Selection Criteria and Posterior Classification Probabilities for Multivariate Latent Class Linear Mixed Modeling (LCMM) of Weight, Depressive Symptoms, Eating Disorder Psychopathology, and Health-Related Quality of Life and for Univariate LCMM of Weight, Longitudinally Conducted over the First Five Years after Obesity Surgery (N=856).

Table S3. Descriptives for Trajectory Variables and Multivariate Trajectory Classes Across 6 Years Following Obesity Surgery (N=856)

Supplemental Figures

Figure S1. Multivariate Trajectory Classes of Low, Medium, and High Sustainability in the Total Sample (N=865), in the Subsample With Available 6-Year Follow-up Assessment (N 225), and in the Complete-Case Subsample (N=164).

Figure S2. Individual Trajectories Within Multivariate Trajectory Classes of Low, Medium, and High Sustainability (N=856).

Figure S3. Individual Trajectories Within Multivariate Trajectory Classes of Low, Medium, and High Sustainability in the Subsample With Available 6-Year Follow-up Assessment (T7, N=225).

Figure S4. Individual Trajectories Within Multivariate Trajectory Classes of Low, Medium, and High Sustainability in the Complete-Case Subsample Subsample (N=164).

Figure S5. Multivariate Trajectory Classes of Low, Medium, and High Sustainability by Surgical Procedure: Roux-en-Y Gastric Bypass (upper; N=567) and Sleeve Gastrectomy (lower; N=289).

Table S1. Correlations Among Trajectory Variables at Baseline (N=856)

	**Weight, kg**	**PHQ-D, 0-27**	**EDE-Q, 0-6**	**IWQOL, 0-100**
**Weight, kg**	-	.02	-.02	-.19
**PHQ-D, 0-27**	.02	-	.56	-.65
**EDE-Q, 0-6**	-.02	.56	-	-.63
**IWQOL, 0-100**	-.19	-.65	-.63	-

Displayed are Spearman's rank correlation coefficients. EDE-Q, Eating Disorder Examination-Questionnaire; IWQOL, Impact of Weight on Quality of Life-Lite; PHQ-D, Patient Health Questionnaire-Depression.

Table S2. Selection Criteria and Class Membership Proportions Based on Posterior Classification Probabilities for Multivariate Latent Class Linear Mixed Modeling (LCMM) of Weight, Depressive Symptoms, Eating Disorder Psychopathology, and Health-Related Quality of Life and for Univariate LCMM of Weight, Longitudinally Conducted over the First Five Years after Obesity Surgery (N=856)

**Multivariate LCMM**								
**N Classes**	**BIC**	**AIC**	**Entropy**	**Class 1**	**Class 2**	**Class 3**	**Class 4**	**Class 5**
2	56095.33	55933.76	0.0075	51.17%	48.83%	-	-	-
**3**	**56076.04**	**55900.20**	**0.6830**	2.8%	89.14%	8.06%	-	-
4	56090.81	55900.72	0.5447	3.50%	5.84%	61.33%	29.32%	-
5	56101.68	55897.33	0.4485	7.94%	9.81%	0	78.39%	3.86%
**Univariate LCMM**								
**N Classes**	**BIC**	**AIC**	**Entropy**	**Class 1**	**Class 2**	**Class 3**	**Class 4**	**Class 5**
2	17848.40	17805.85	0.0008	48.62%	51.38%	-	-	-
**3**	**17840.39**	**17783.66**	**0.9553**	0.24%	98.92%	0.84%	-	-
4	17859.78	17788.87	0.8090	0.24%	98.2%	0.72%	0.84%	-
5	17872.65	17787.56	0.6755	0.24%	87.78%	2.75%	8.50%	0.72%

The models with most favorable selection criteria are bolded. Selection criteria: BIC, Bayesian information criterion; AIC, Akaike information criterion; entropy. LCMM variables included relative weight loss (i.e., percentage total body weight loss) from baseline and absolute change scores from baseline for eating disorder psychopathology (Eating Disorder Examination-Questionnaire global score, 0-6), health-related quality of life (Impact of Weight on Quality of Life-Lite total score, 0-100), depressive symptoms (Patient Health Questionnaire-Depression score, 0-27).

Table S3. Descriptives for Multivariate Trajectory Variables and Classes Across 6 Years Following Obesity Surgery

		T0		T2		T3		T4	
		**M (SD)**	**N**	**M (SD)**	**N**	**M (SD)**	**N**	**M (SD)**	**N**
**Weight, kg**	**All**	**141.74 (28.68)**	**856**	**101.28 (24.78)**	**754**	**99.36 (24.07)**	**629**	**101.65 (24.62)**	**533**
Class	1	140.50 (25.46)	24	99.16 (17.54)	23	105.77 (20.14)	20	104.68 (16.34)	18
	2	141.15 (28.40)	763	101.29 (24.93)	673	99.93 (24.43)	554	102.61 (25.09)	464
	3	148.78 (32.09)	69	102.04 (25.72)	58	91.26 (19.85)	55	91.87 (20.53)	51
**IWQOL, 0-100**	**All**	**48.29 (21.83)**	**778**	**83.17 (17.96)**	**695**	**85.12 (17.57)**	**618**	**84.82 (16.68)**	**503**
Class	1	41.12 (19.91)	24	82.82 (10.98)	23	70.55 (22.89)	21	66.82 (22.65)	20
	2	50.89 (21.14)	685	83.42 (18.13)	605	85.20 (17.59)	542	84.90 (16.57)	463
	3	24.87 (13.08)	69	81.08 (18.44)	67	89.91 (11.26)	55	90.87 (8.61)	53
**PHQ-D, 0-27**	**All**	**7.75 (5.14)**	**777**	**4.69 (4.11)**	**696**	**5.14 (4.54)**	**623**	**5.35 (4.57)**	**541**
Class	1	8.21 (5.30)	24	5.87 (3.22)	23	8.62 (5.82)	21	8.83 (5.24)	21
	2	7.26 (4.93)	684	4.64 (4.11)	606	5.07 (4.55)	546	5.27 (4.57)	466
	3	12.49 (4.70)	69	4.79 (4.32)	67	4.55 (3.38)	56	4.63 (3.75)	54
**EDE-Q, 0-6**	**All**	**2.92 (1.01)**	**779**	**1.55 (1.13)**	**695**	**1.48 (1.23)**	**621**	**1.51 (1.25)**	**537**
Class	1	3.08 (0.96)	24	1.90 (1.24)	23	2.49 (1.51)	21	2.48 (1.40)	21
	2	2.82 (0.98)	686	1.54 (1.14)	605	1.48 (1.23)	544	1.51 (1.23)	462
	3	3.88 (0.84)	69	1.45 (1.07)	67	1.14 (0.89)	56	1.13 (1.18)	54

Table S3 (cont.)

		T5		T6		T7	
		M (SD)	N	M (SD)	N	M (SD)	N
**Weight, kg**	**All**	**103.28 (25.22)**	**438**	**104.54 (23.27)**	**329**	**104.06 (23.11)**	**225**
Class	1	111.78 (23.55)	17	115.94 (21.73)	14	113.42 (22.04)	10
	2	103.81 (25.47)	386	105.26 (23.05)	282	105.16 (23.06)	222
	3	93.34 (20.77)	35	93.56 (22.54)	33	89.34 (18.65)	23
**IWQOL, 0-100**	**All**	**83.99 (17.74)**	**431**	**82.17 (19.17)**	**302**	**80.83 (20.55)**	**224**
Class	1	58.86 (20.34)	19	44.80 (13.06)	15	53.36 (25.85)	9
	2	84.50 (17.16)	372	83.78 (17.78)	257	80.92 (19.93)	192
	3	92.51 (7.40)	40	95.14 (5.54)	30	90.76 (13.33)	23
**PHQ-D, 0-27**	**All**	**5.48 (4.69)**	**434**	**6.31 (5.26)**	**303**	**6.30 (5.53)**	**223**
Class	1	9.67 (3.84)	21	14.13 (5.11)	15	12.33 (6.42)	9
	2	5.40 (4.74)	373	6.13 (5.08)	258	6.19 (5.53)	191
	3	4.03 (3.31)	40	3.97 (2.99)	30	4.83 (3.45)	23
**EDE-Q, 0-6**	**All**	**1.44 (1.21)**	**435**	**1.58 (1.39)**	**301**	**1.65 (1.32)**	**225**
Class	1	2.90 (1.29)	21	3.65 (1.15)	15	3.70 (1.58)	9
	2	1.45 (1.18)	374	1.56 (1.33)	256	1.60 (1.26)	193
	3	0.64 (0.68)	40	0.67 (0.74)	30	1.26 (0.96)	23

IWQOL, Impact of Weight on Quality of Life-Lite; PHQ-D, Patient Health Questionnaire-Depression; EDE-Q, Eating Disorder Examination-Questionnaire. Class1, *low sustainability*; class 2, *medium sustainability*; class 3, *high sustainability*.

Figure S1. Multivariate Trajectory Classes of Low, Medium, and High Sustainability in the Total Sample (N=865), in the Subsample With Available 6-Year Follow-up Assessment (N=225), and in the Complete-Case Subsample (N=164).

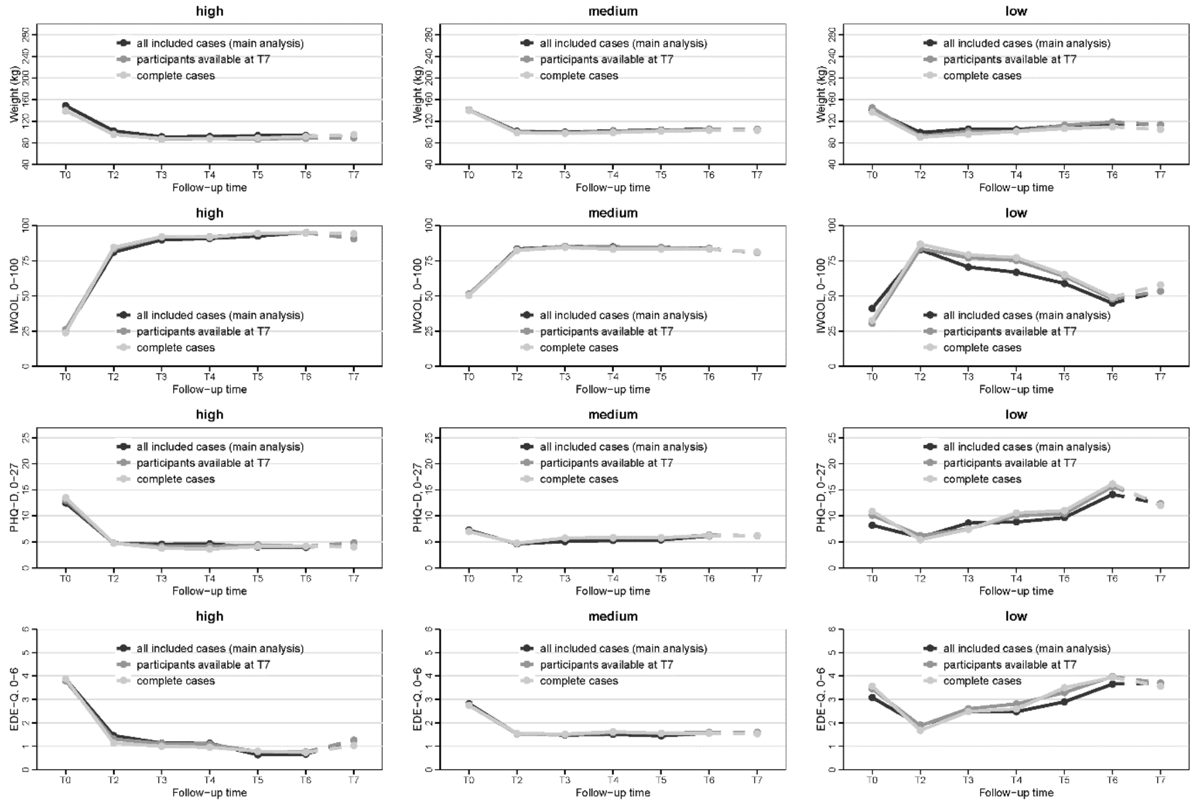

Note. Subsample with available 6-year follow-up (T7) data: *low sustainability*: 10.37% (17/164), *medium sustainability*: 85.37% (140/164), *high sustainability*: 4.27% (7/140); complete-case subsample: *low sustainability*: 10.22% (23/225), *medium sustainability*: 85.78% (193/225), *high sustainability*: 4.00% (4/225).

Figure S2. Individual Trajectories Within Multivariate Trajectory Classes of Low, Medium, and High Sustainability (N=856).

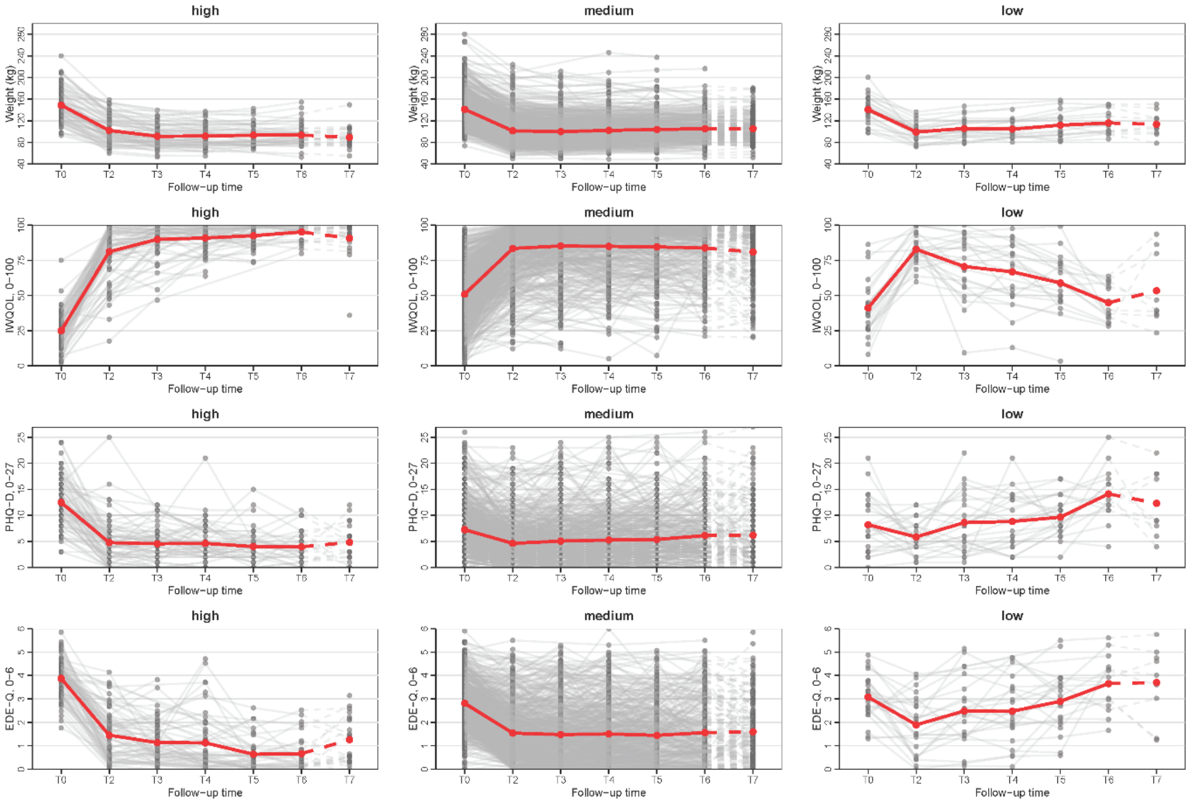

Figure S3. Individual Trajectories Within Multivariate Trajectory Classes of Low, Medium, and High Sustainability in the Subsample With Available 6-Year Follow-up Assessment (T7, N=225).

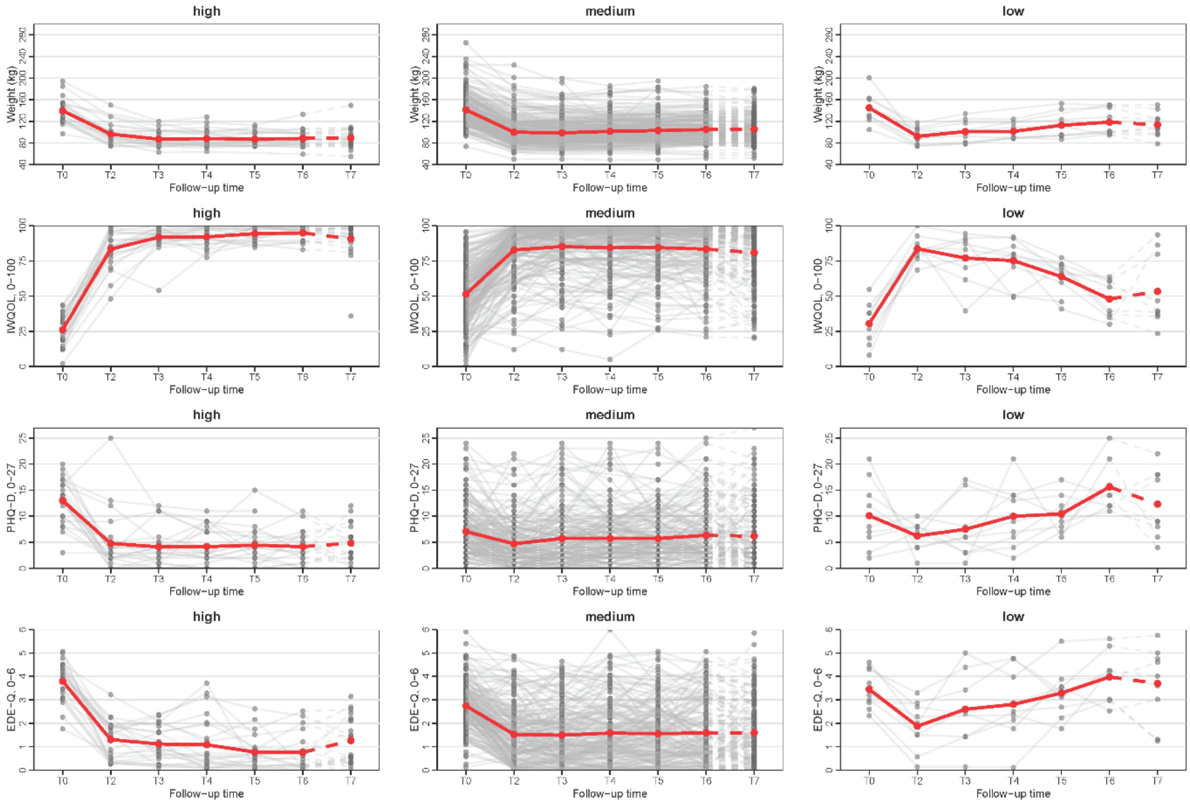

Figure S4. Individual Trajectories Within Multivariate Trajectory Classes of Low, Medium, and High Sustainability in the Complete-Case Subsample (N=164).

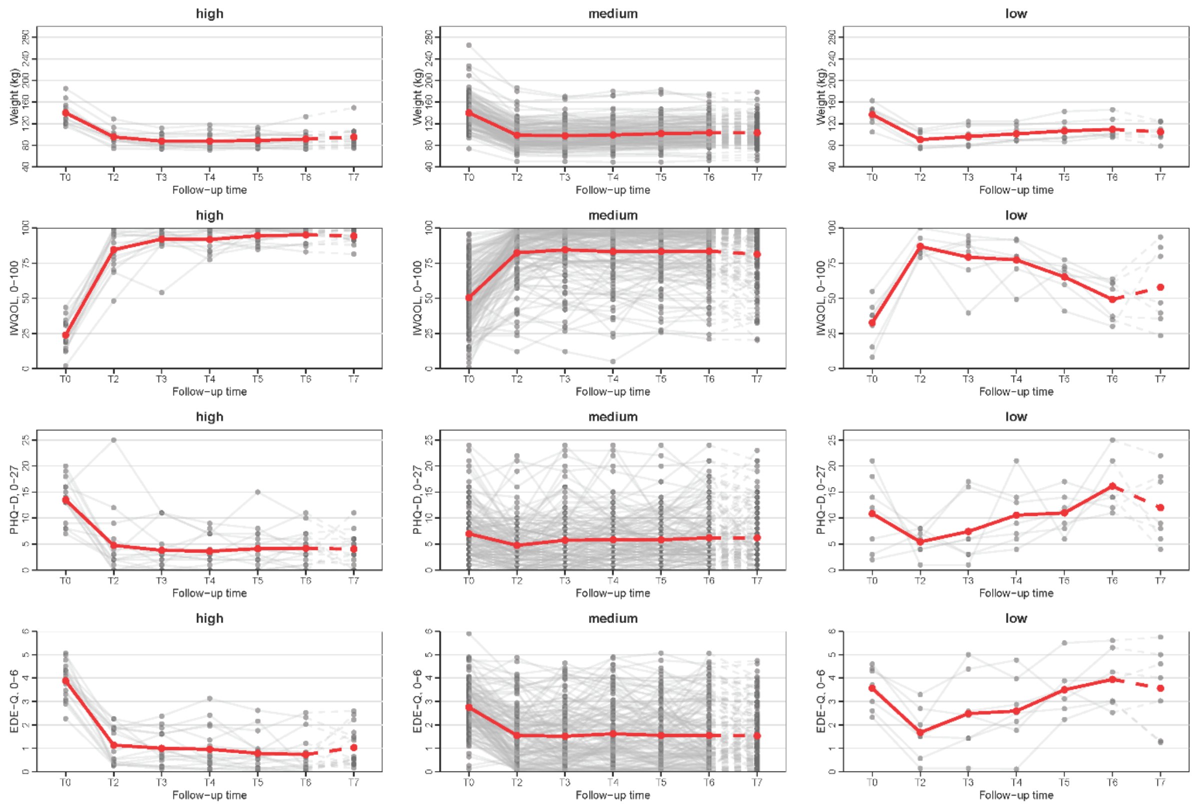

Figure S5. Multivariate Trajectory Classes of Low, Medium, and High Sustainability by Surgical Procedure: (I) Roux-en-Y Gastric Bypass (N=567) and (II) Sleeve Gastrectomy (N=289).

I Roux-en-Y Gastric Bypass

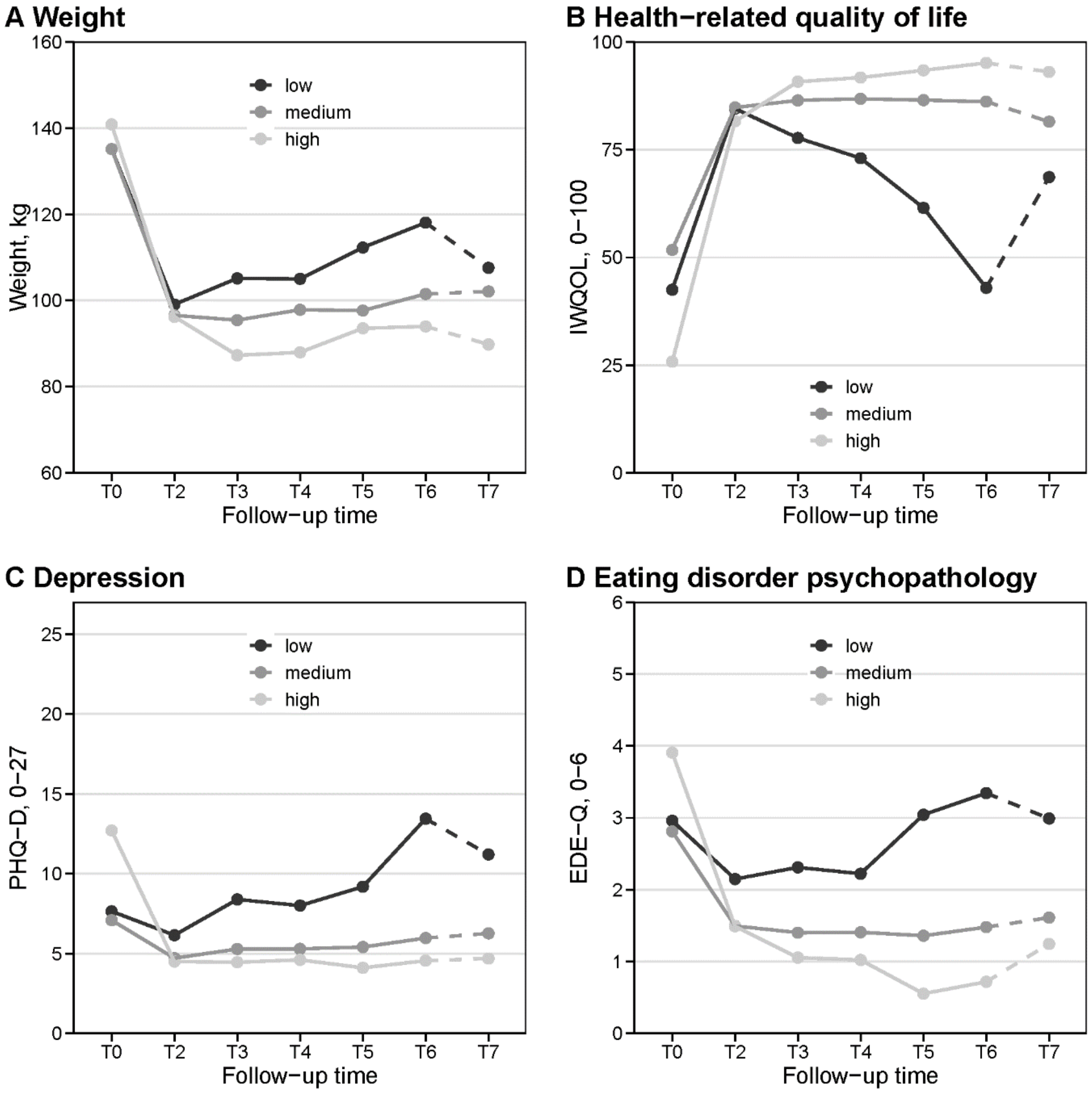

II Sleeve Gastrectomy

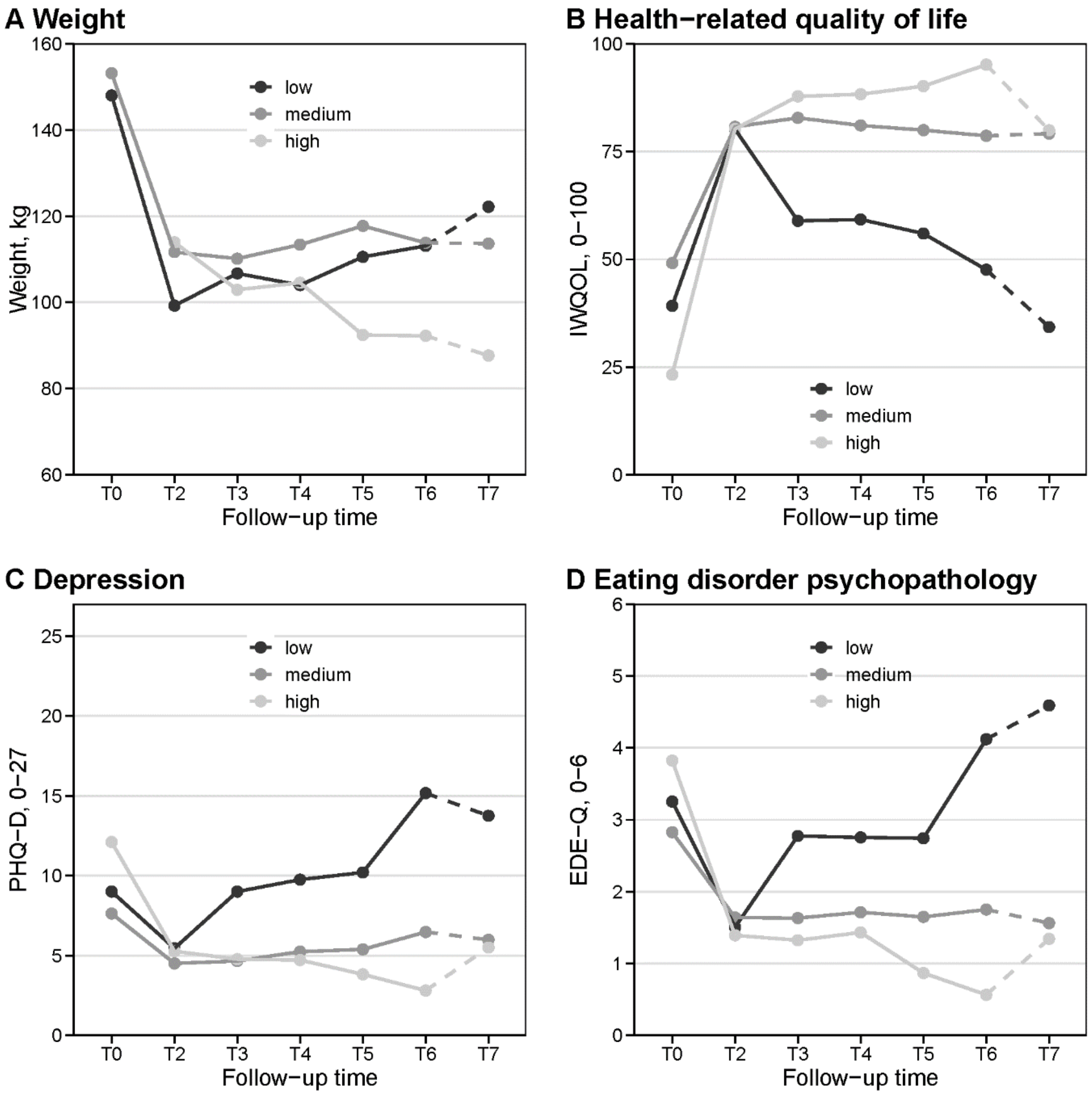

Displayed are means from valid cases. The trajectory classes were determined using Latent Class Linear Mixture Modeling in the total sample (N=856) from baseline through the first five years after obesity surgery (T0-T6) and applied to the 6-year follow-up (T7). EDE-Q, Eating Disorder Examination-Questionnaire; IWQOL, Impact of Weight on Quality of Life-Lite; PHQ-D, Patient Health Questionnaire-Depression.
